# What is the benefit of inconsistent opioid agonist treatment in patients with prescription opioid use disorder?

**DOI:** 10.1192/j.eurpsy.2023.1196

**Published:** 2023-07-19

**Authors:** R. Weiss, M. L. Griffin

**Affiliations:** Psychiatry/Division of Alcohol, Drugs, and Addiction, Harvard Medical School/McLean Hospital, Belmont, MA, United States

## Abstract

**Introduction:**

Studies consistently show that patients with prescription opioid use disorder (OUD) respond to buprenorphine treatment. Few studies have followed these patients in the long-term. Our longitudinal research has shown opioid abstinence to be associated most strongly with opioid agonist/partial agonist treatment. We also found that many patients used agonist treatment inconsistently; questions remain about the benefits of intermittent opioid agonist treatment.

**Objectives:**

We examined patients during the 3.5 years following their entry into a 3-month trial of treatment for prescription OUD. The current analysis compared opioid use outcomes among patients who reported receipt of agonist treatment consistently, inconsistently, or never.

**Methods:**

This secondary analysis (N=309) of a U.S. multi-site randomized controlled trial of treatment for prescription OUD assessed variability in receiving opioid agonist treatment during the 3.5-year follow-up period, and the association between agonist treatment and opioid abstinence. Assessments were collected at months 18, 30, and 42 following treatment entry; patients were asked if they were currently taking agonist treatment and whether they had used other opioids in the previous month. Patients with only one follow-up assessment (n=29) were excluded from this analysis.

**Results:**

Most patients reported current opioid abstinence on at least one follow-up visit: 38% were always abstinent, 41% sometimes, and 21% never. Twenty-three percent always reported currently using agonist treatment, 26% sometimes, and 51% never. Patients consistently reporting agonist use were most likely to always be opioid-abstinent in the past month (69%), with 25% sometimes and 6% never abstinent. Patients who never reported agonist use were equally likely to be abstinent never (32%), sometimes (35%), and always (32%). Patients who sometimes reported receiving agonists were most likely to report abstinence sometimes (65%); 14% never reported abstinence, and 21% always did.

Those consistently receiving agonist treatment were more likely to always be opioid-abstinent (69%) than those sometimes (21%) or never (32%) receiving agonists. Those never receiving agonist treatment were more likely to never report opioid abstinence (32%) than were those sometimes (14%) or always (6%) receiving agonists. Interestingly, those who sometimes received agonists were more likely to be abstinent than those who never received agonists: those who sometimes received agonists were more likely to be abstinent sometimes than those who never received agonists (65% vs. 35%) and less likely to never be abstinent than were those who never received agonists (14% vs. 32%).

**Conclusions:**

Receiving opioid agonist treatment has been shown to be associated with opioid abstinence during long-term follow-up. This study shows that even those who only inconsistently receive agonists are also likely to benefit.

**Disclosure of Interest:**

R. Weiss Consultant of: Alkermes, M. Griffin: None Declared

